# Supplementary material for: Species abundance information improves sequence taxonomy classification accuracy
Source: Nat Commun. 2019 Oct 11;10:4643. doi: 10.1038/s41467-019-12669-6 (PMC6789115; doi:10.1038/s41467-019-12669-6)
Supplement: Supplementary file 1 — Supplementary Information [file 41467_2019_12669_MOESM1_ESM.pdf]

Species abundance information improves sequence taxonomy classification accuracy

Kaehler et al.

Supplementary Figures

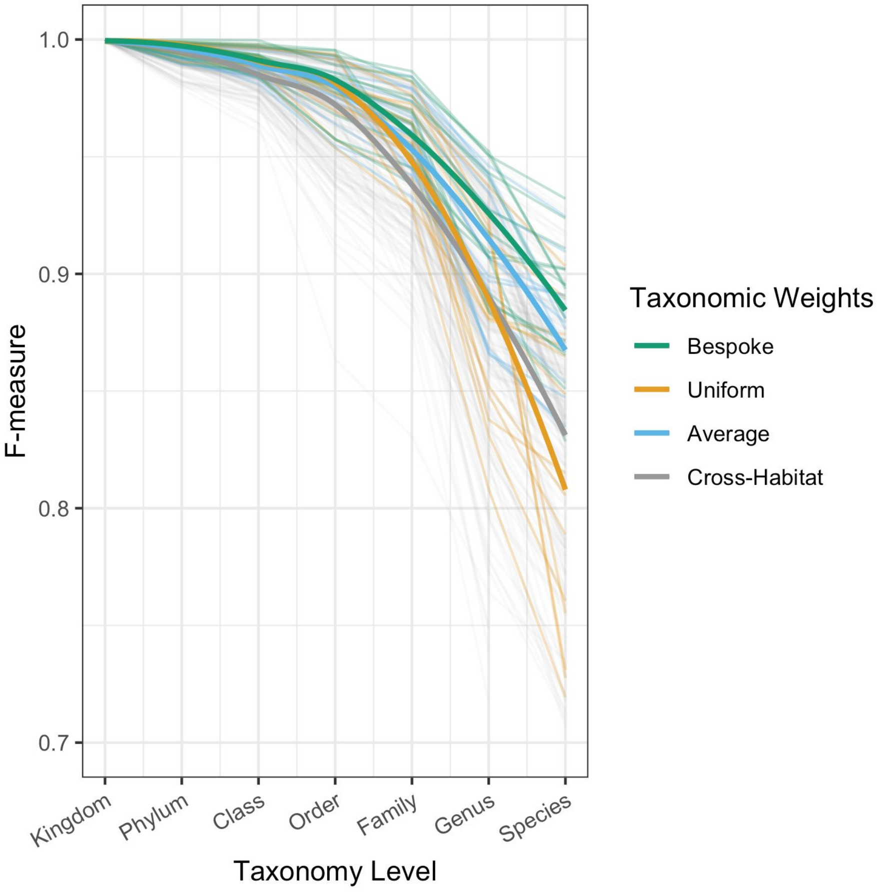

Supplementary Figure 1**.** Classification accuracy drops with increasing taxonomic specificity. F-measures are for 5-fold cross validation where classifiers trained using a variety of taxonomic weighting strategies are tested on sequences and empirical taxonomic abundances that were not used in training. Classification F-measure drops as finer levels of classification are required, but is much more consistent across levels for classifiers with bespoke (habitat-specific) weights. Bespoke weights were habitat-specific. Average weights were averaged across the 14 EMPO 3 habitats. Uniform weights are the current best practice. Cross-habitat weights were weights from EMPO 3 habitats other than the sample's habitat. Faint lines show results for 14 EMPO 3 habitat types. Bold lines show LOESS plots to demonstrate trends. Source data are provided as a Source Data file.

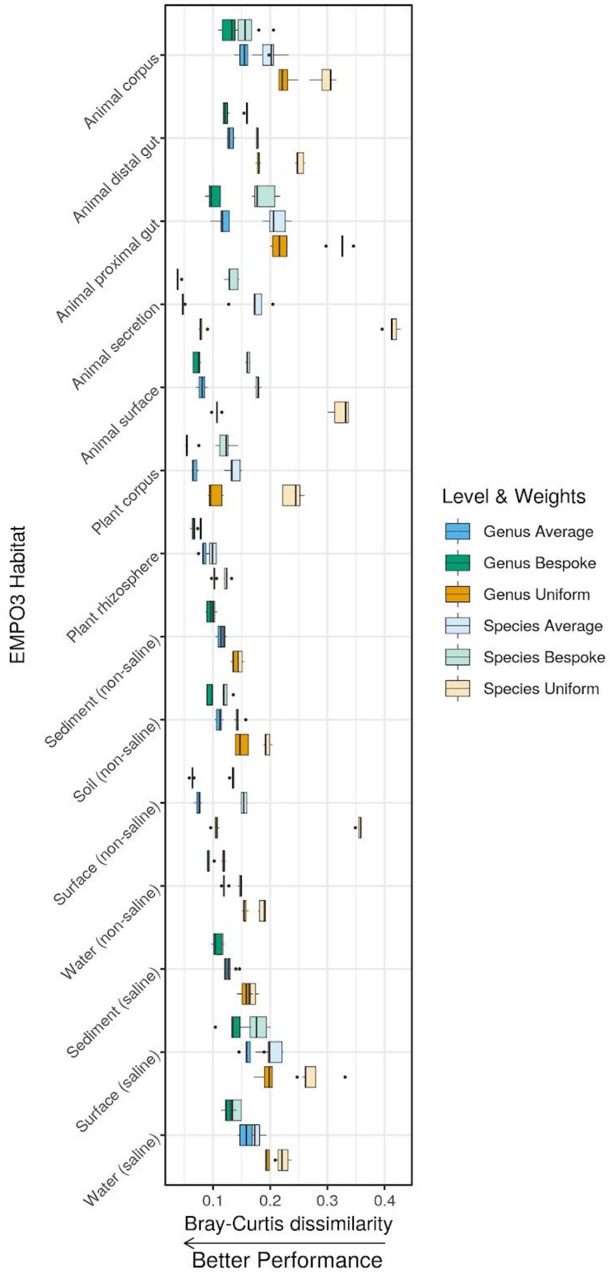

Supplementary Figure 2**.** Bespoke weights always outperformed average weights across EMPO 3 habitat types, and average weights always outperformed uniform weights (sign test P = $6.1\text{x}10^{-5}$). Plot shows Bray-Curtis dissimilarity between expected and observed taxonomic abundances for differing taxonomic weighting strategies and at genus and species levels. Bespoke weights were habitat-specific. Average weights were averaged across the 14 EMPO 3 habitats. Uniform weights are the current best practice. Tests were based on 5-fold cross validation across 18,222 empirical taxonomic abundances. Box plots are across folds. Box bounds and centre lines show first and third quartiles and median. Whiskers extend to measurements no further than 1.5 times the interquartile range from the nearest quartiles. Outliers are plotted individually. Source data are provided as a Source Data file.

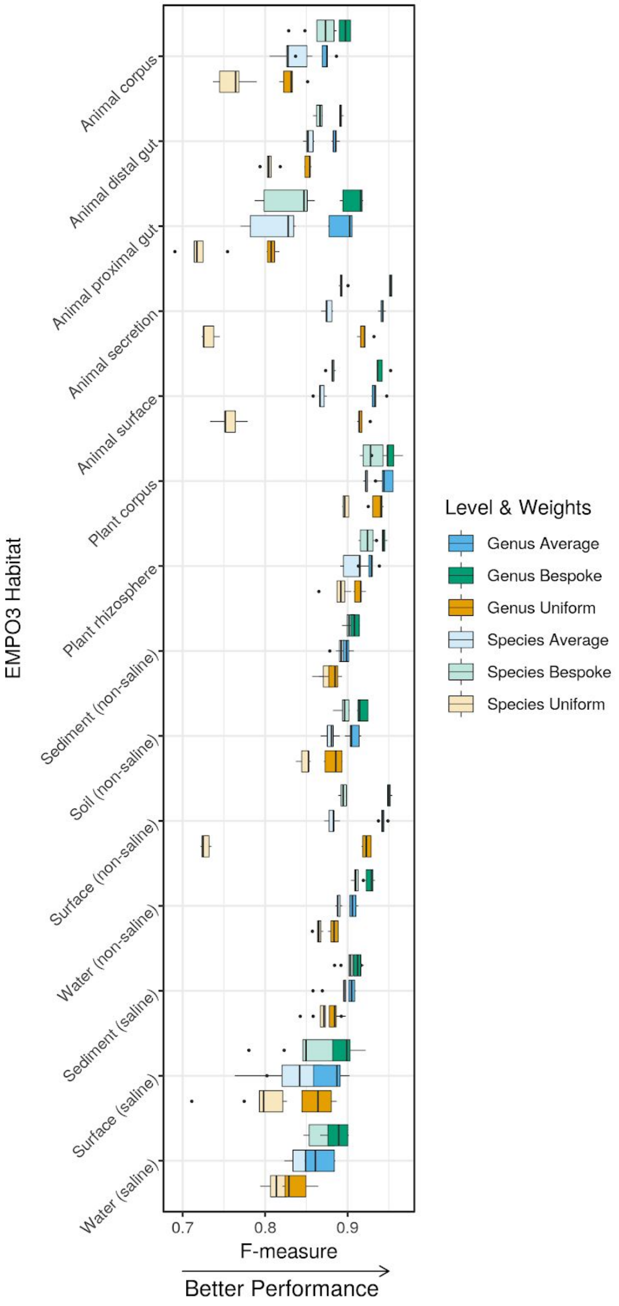

Supplementary Figure 3**.** Bespoke weights always outperformed average weights across EMPO 3 habitat types, and average weights always outperformed uniform weights at both the genus and species levels (sign test $P = 6.1 \times 10^{-5}$)**.** F-measures are from 5-fold cross validation where classifiers trained using a variety of taxonomic weighting strategies are tested on sequences and empirical taxonomic abundances that were not used in training. Tests were based on 21,513 empirical samples across the 14 habitat types. Box bounds and centre lines show first and third quartiles and median. Whiskers extend to measurements no further than 1.5 times the interquartile range from the nearest quartiles. Outliers are plotted individually. Source data are provided as a Source Data file.

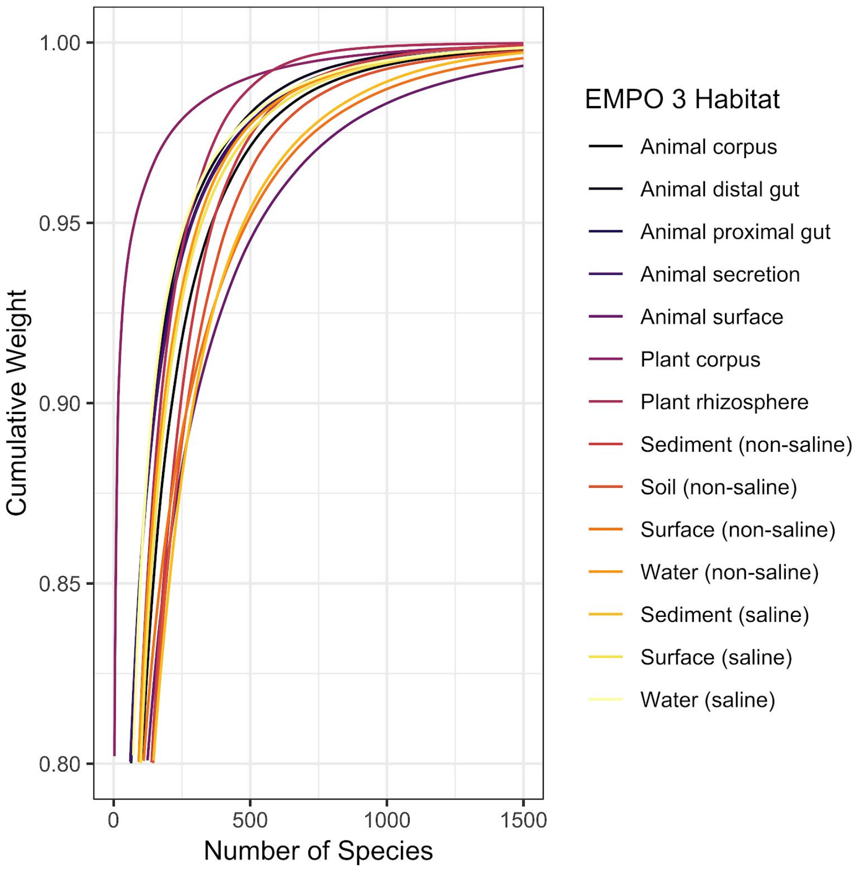

Supplementary Figure 4**.** Cumulative weights for different habitat types display similar diversity trends. Lines show the cumulative taxonomic weights for 14 EMPO 3 habitat types as a function of species count, coloured by habitat. Taxa are ordered separately for each habitat from most to least abundant. The most peaked distribution is Plant corpus, where 73% of reads were mapped to a single taxonomy in the Cyanobacteria phylum, Chloroplast class. Source data are provided as a Source Data file.

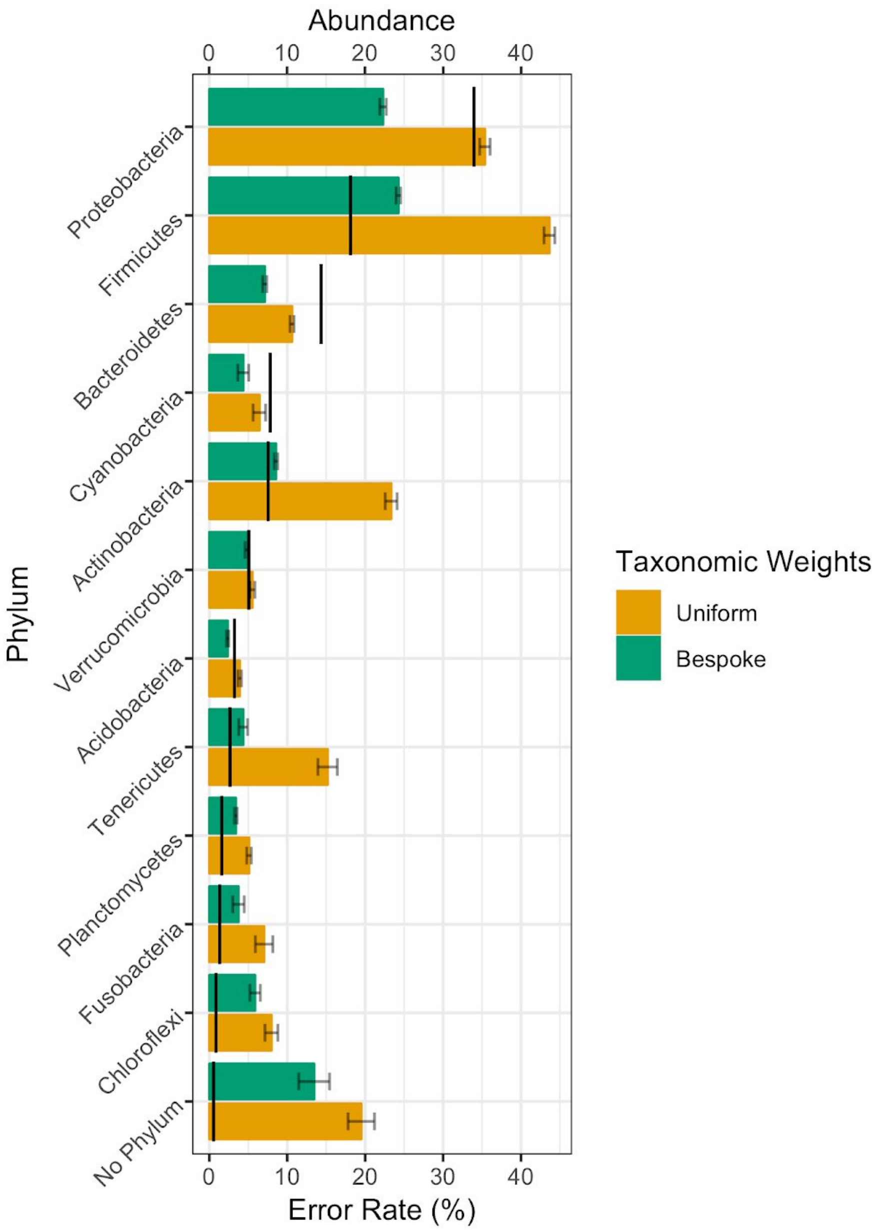

Supplementary Figure 5**.** Habitat-specific taxonomic weights improve species-level classification accuracy across phyla. The use of habitat-specific weights is more important for species classification within some phyla, but is more important for more abundant phyla. Columns show percentage of reads correctly classified averaged across 14 EMPO 3 habitats and 21,513 empirical samples. Black lines show average abundances for each phylum. The phyla were truncated to only show those with an average abundance of > 0.05%. Error bars show standard error. Source data are provided as a Source Data file.

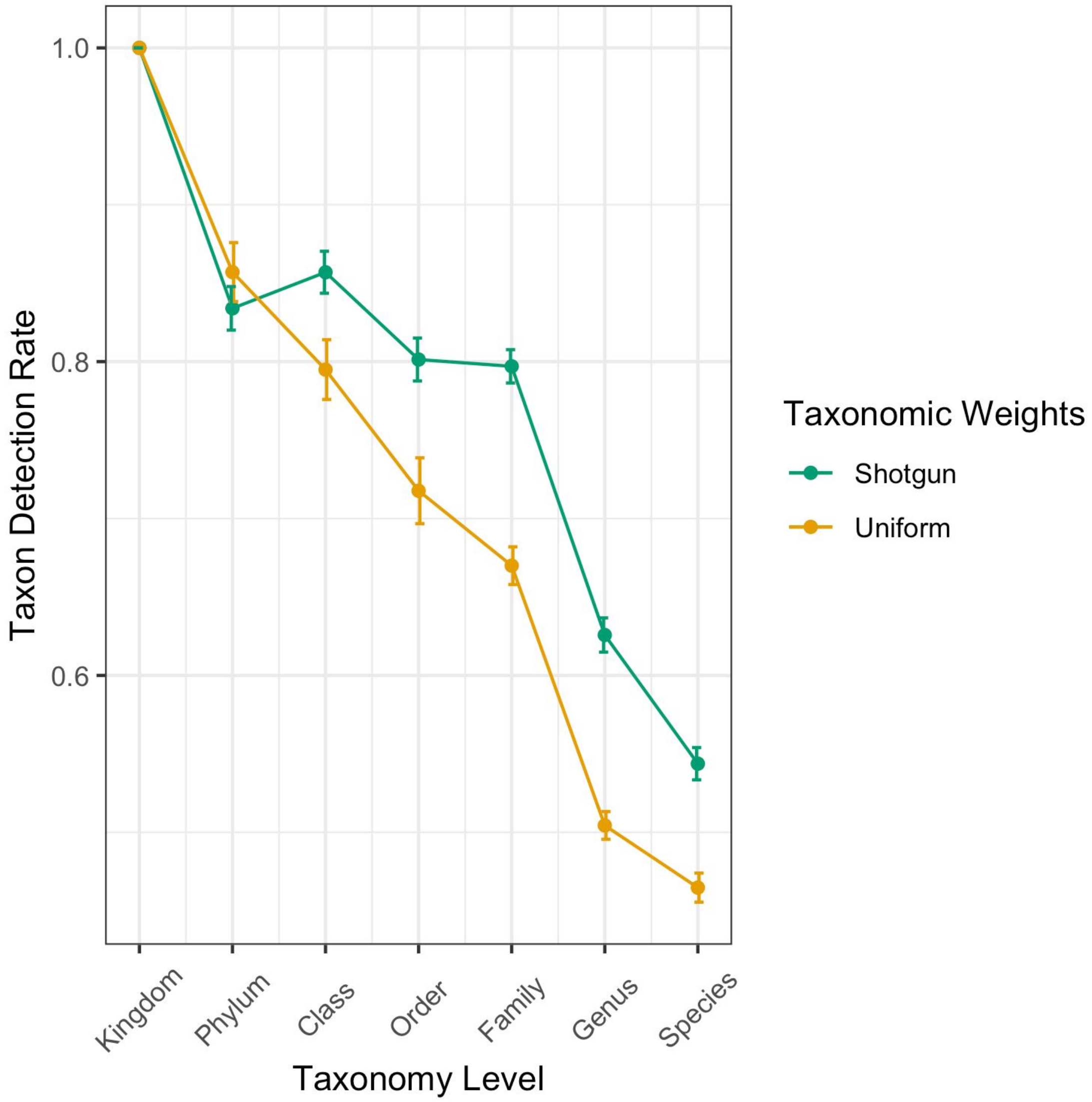

Supplementary Figure 6**.** Taxonomic weights from shotgun sequencing improve agreement between amplicon and shotgun sequencing taxonomic compositions. Taxonomic weights derived from shotgun sequencing experiments make the taxa discovered in out-of-sample 16S sequencing samples agree more closely with shotgun sequencing experiments on the same samples. Taxon detection rate (TDR) is the fraction of taxa detected using shotgun sequencing that were also found using 16S sequencing. Points show mean TDR across 71 stool samples for which paired 16S and shotgun sequencing exists. Error bars show standard errors across folds for 5-fold cross validation. Source data are provided as a Source Data file.

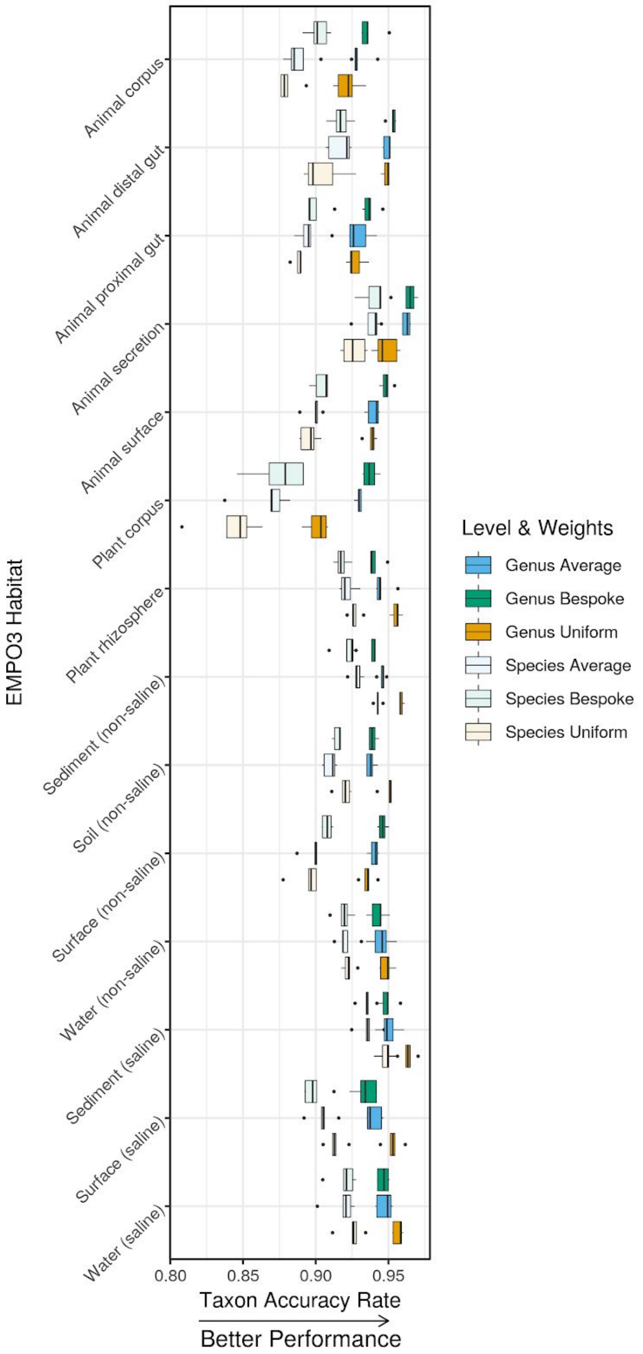

Supplementary Figure 7**.** On average, bespoke weights outperformed average weights across EMPO 3 habitat types, and average weights outperformed uniform weights based on Taxon Accuracy Rate (but neither significantly, minimum paired t-test $P = 0.17$)**.** Taxon Accuracy Rates are from 5-fold cross validation where classifiers were trained using a variety of taxonomic weighting strategies. Tests were based on 21,513 empirical samples across the 14 habitat types. Box bounds and centre lines show first and third quartiles and median. Whiskers extend to measurements no further than 1.5 times the interquartile range from the nearest quartiles. Outliers are plotted individually. Source data are provided as a Source Data file.

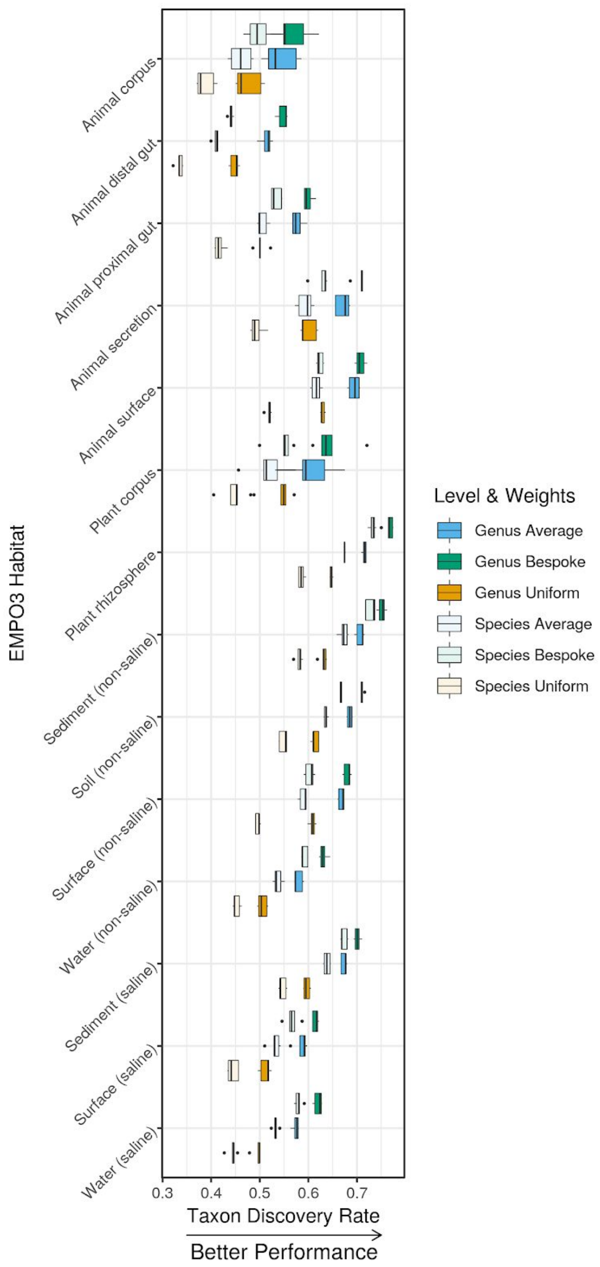

Supplementary Figure 8. Bespoke weights always outperformed average weights across EMPO 3 habitat types, and average weights always outperformed uniform weights based on Taxon Detection Rate (maximum paired t-test *P* = 4.7x10-7). Taxon Detection Rates are from 5-fold cross validation where classifiers were trained using a variety of taxonomic weighting strategies. Tests were based on 21,513 empirical samples across the 14 habitat types. Box bounds and centre lines show first and third quartiles and median. Whiskers extend to measurements no further than 1.5 times the interquartile range from the nearest quartiles. Outliers are plotted individually. Source data are provided as a Source Data file.

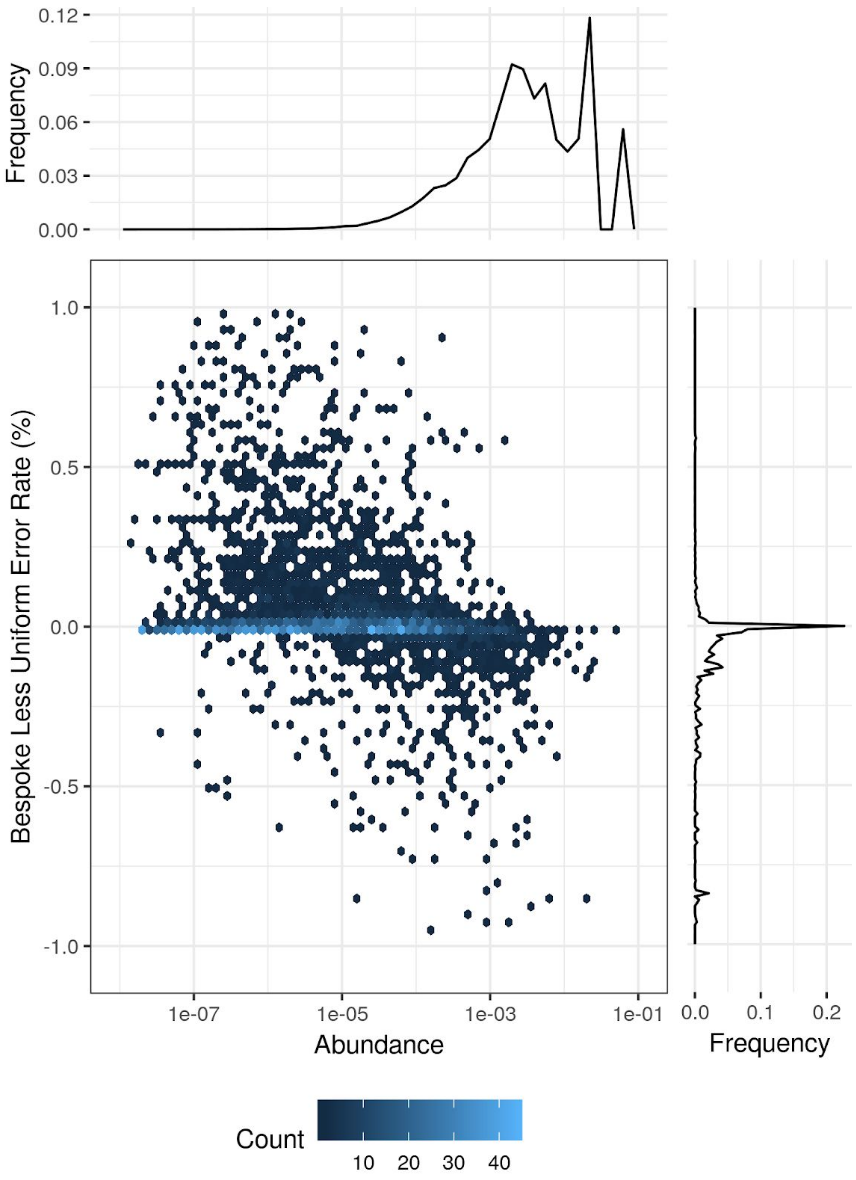

Supplementary Figure 9**.** Error rates improve less for less abundant species than for more abundant species under bespoke weights. Hex bin plot shows difference in error rates between bespoke and uniform weighting strategies, averaged over each species, then across all the samples for each of the 14 EMPO 3 habitat types, then across the habitat types. Histograms show abundances averaged in the same way then marginalised. Whereas the hex bin plot indicates the presence of rare ASVs that are more accurately classified under uniform weights, the histograms indicate that bespoke weights provide at least a slight improvement for the vast majority of ASVs detected across all sample types. Source data are provided as a Source Data file.

Supplementary Tables

Supplementary Table 1**.** Using habitat-specific taxonomic weights, researchers can now classify sequences at species level with the same confidence that they previously classified sequences at genus level**.** Table shows error rates at genus and species levels for habitat-specific (bespoke) and standard (uniform) taxonomic weights. Bolded rows indicate EMPO 3 habitats where species-level error rate with the bespoke classifier is less than genus-level accuracy with the uniform classifier. Lower error rate indicates superior accuracy. Source data are provided as a Source Data file.

	Error Rate (%)*			
	Bespoke		Uniform	
EMPO 3 Habitat	Genus	Species	Genus	Species
**Animal corpus**	**14.1**	**16.6**	**21.7**	**30.3**
**Animal distal gut**	**13.5**	**16.3**	**18.3**	**23.9**
**Animal proximal gut**	**11.2**	**21.3**	**24.6**	**35.0**
Animal secretion	6.2	14.5	10.9	36.1
Animal surface	7.7	16.2	11.4	33.0
Plant corpus	5.7	8.2	8.0	12.8
**Plant rhizosphere**	**7.7**	**9.7**	**11.8**	**14.9**
**Sediment (non-saline)**	**11.8**	**12.3**	**15.2**	**15.9**
**Soil (non-saline)**	**10.8**	**13.3**	**15.9**	**20.1**
Surface (non-saline)	6.5	14.4	10.9	37.4
**Water (non-saline)**	**9.9**	**11.8**	**15.8**	**17.9**
**Sediment (saline)**	**11.5**	**12.1**	**15.2**	**16.6**
**Surface (saline)**	**14.8**	**17.9**	**19.4**	**26.6**
**Water (saline)**	**14.5**	**15.7**	**20.3**	**22.9**

*Error rates (proportion of reads incorrectly classified) are from 5-fold cross validation where classifiers were tested on sequences and empirical taxonomic abundances that were not used in training. Tests were based on 21,513 empirical samples across the 14 habitat types.

Supplementary Table 2. Using habitat-specific taxonomic weights, researchers can now classify sequences at species level with the same confidence that they previously classified sequences at genus level. Table shows Bray-Curtis dissimilarity at genus and species levels for habitat-specific (bespoke) and standard (uniform) taxonomic weights. Bolded rows indicate EMPO 3 habitats where species-level Bray-Curtis dissimilarity with the bespoke classifier is less than genus-level accuracy with the uniform classifier. Lower Bray-Curtis dissimilarity indicates superior accuracy. Source data are provided as a Source Data file.

	Bray-Curtis Dissimilarity*			
	Bespoke		Uniform	
EMPO 3 Habitat	Genus	Species	Genus	Species
**Animal corpus**	**0.13**	**0.15**	**0.21**	**0.29**
**Animal distal gut**	**0.11**	**0.13**	**0.16**	**0.21**
**Animal proximal gut**	**0.10**	**0.20**	**0.24**	**0.34**
Animal secretion	0.05	0.13	0.10	0.35
Animal surface	0.07	0.14	0.11	0.32
**Plant corpus**	**0.06**	**0.08**	**0.08**	**0.13**
**Plant rhizosphere**	**0.06**	**0.08**	**0.11**	**0.14**
**Sediment (non-saline)**	**0.09**	**0.10**	**0.13**	**0.14**
**Soil (non-saline)**	**0.09**	**0.11**	**0.15**	**0.19**
Surface (non-saline)	0.06	0.13	0.10	0.36
**Water (non-saline)**	**0.09**	**0.11**	**0.15**	**0.17**
**Sediment (saline)**	**0.09**	**0.10**	**0.13**	**0.15**
**Surface (saline)**	**0.13**	**0.16**	**0.18**	**0.25**
**Water (saline)**	**0.13**	**0.14**	**0.19**	**0.22**

*Bray-Curtis dissimilarities are from 5-fold cross validation where classifiers were tested on sequences and empirical taxonomic abundances that were not used in training. Tests were based on 18,222 empirical samples across the 14 habitat types.

Supplementary Table 3. Using habitat-specific taxonomic weights, researchers can now classify sequences at species level with the same confidence that they previously classified sequences at genus level. Table shows F-measure at genus and species levels for habitat-specific (bespoke) and standard (uniform) taxonomic weights. Bolded rows indicate EMPO 3 habitats where species-level F-measure with the bespoke classifier is greater than genus-level accuracy with the uniform classifier. Greater F-measure indicates superior accuracy. Source data are provided as a Source Data file.

	F-measure*			
	Bespoke		Uniform	
EMPO 3 Habitat	Genus	Species	Genus	Species
**Animal corpus**	**0.89**	**0.87**	**0.83**	**0.76**
**Animal distal gut**	**0.89**	**0.87**	**0.85**	**0.81**
**Animal proximal gut**	**0.91**	**0.83**	**0.81**	**0.72**
Animal secretion	0.95	0.89	0.92	0.73
Animal surface	0.94	0.88	0.92	0.76
Plant corpus	0.95	0.93	0.94	0.90
**Plant rhizosphere**	**0.94**	**0.92**	**0.91**	**0.89**
**Sediment (non-saline)**	**0.91**	**0.90**	**0.88**	**0.87**
**Soil (non-saline)**	**0.92**	**0.90**	**0.88**	**0.85**
Surface (non-saline)	0.95	0.89	0.92	0.73
**Water (non-saline)**	**0.93**	**0.91**	**0.88**	**0.86**
**Sediment (saline)**	**0.91**	**0.90**	**0.88**	**0.87**
**Surface (saline)**	**0.89**	**0.85**	**0.85**	**0.79**
**Water (saline)**	**0.89**	**0.87**	**0.84**	**0.81**

*F-measures are for 5-fold cross validation where classifiers were tested on sequences and empirical taxonomic abundances that were not used in training. Tests were based on 21,513 empirical samples across the 14 habitat types.

Supplementary Notes

Using cross validation (see Methods), we determined the effect of several different options for obtaining taxonomic weights on taxonomic classification accuracy. We labelled those options as:

- Uniform weights: every taxonomic class is assumed to be equally likely.
- Bespoke weights: weights drawn from the same EMPO 3 habitat as the test samples.
- Cross-habitat weights: weights from any of the 13 EMPO 3 habitats other than a test sample's source EMPO 3 habitat.
- Average weights: weights obtained by averaging across all 14 EMPO 3 habitats.

Uniform weights is the current default assumption for q2-feature-classifier and the only available option for the RDP Classifier. Average weights were used to determine how important it is to closely match the taxonomic weights with the expected weights for a given sample, and to investigate a classification approach for uncharacterized and unknown sample types. Cross-habitat weights were used to determine the effect of classifying reads using misspecified weights. For example, if one were to take a sample that would properly be labelled as Animal distal gut, but erroneously undertake taxonomic classification using a classifier trained using Plant corpus weights.

We used three measures of classification accuracy: error rate, Bray-Curtis dissimilarity, and F-measure, made possible because for each test sample there is a known taxonomy for each read. Please refer to the Methods for details of how these measures were calculated and averaged across samples.

As is typical for existing taxonomy classification methods, classification accuracy was excellent at class level, but decreased at finer levels of taxonomic resolution (Supplementary Figure 1). Classification accuracy decreased for both bespoke and uniform weighted classifiers, but bespoke classifiers were much less prone to this effect (Supplementary Figure 1). For uniform weights, the mean F-measure across 14 EMPO 3 habitat types was 0.992 (0.001 standard error), 0.88 (0.01 standard error), 0.81 (0.02 standard error) at class, genus, and species levels, respectively. For bespoke weights, the mean F-measure was 0.992 (0.001 standard error), 0.92 (0.01 standard error), 0.89 (0.01 standard error) at class, genus, and species levels, respectively.

A direct comparison between classification accuracy using bespoke weights versus uniform weights across the 14 EMPO 3 habitat types is given in Supplementary Tables 1-3, Figure 2, and Supplementary Figures 2-3 for error rate, F-measure, and Bray-Curtis dissimilarity. In all cases, classification accuracy was better for bespoke weights at species level than for uniform weights at genus level for 10 of the EMPO 3 habitat types (although these 10 habitat types differ among the three measures). The mean error rate (the proportion of reads incorrectly classified) across the 14 EMPO 3 habitat types was 14% (1% standard error) for bespoke weights at species level and 16% (1% standard error) for uniform weights at genus level (single-sided paired t-test $P = 0.14$) (Figure 1). These results indicate that bespoke weights achieve comparable or better species-level accuracy to what uniform weights can only accomplish at genus level. The mean Bray-Curtis dissimilarity for bespoke weights at species level (0.126, 0.009 standard error) is less than that for uniform weights at genus level (0.15, 0.01 standard error), indicating greater classification accuracy, with a single-sided paired t test $P = 0.013$. The

mean F-measure for bespoke weights at species level (0.887, 0.007 standard error) exceeds that for uniform weights at genus level (0.88, 0.01 standard error) and fails to reject that they are different under a paired t test at 5% significance (P = 0.28). These results verify our claim that on average, classification accuracy at species level for bespoke weights matches or exceeds that for uniform weights at genus level.

Taxonomic classification was tested for uniform, bespoke, average, and cross-habitat taxonomic weights across 14 EMPO 3 habitat types for classification at species level. The results for uniform, bespoke, and average weights are shown for error rate, Bray-Curtis dissimilarity, and F-measure in Figure 2, Supplementary Figure 2, and Supplementary Figure 3, respectively. F-measures for cross-habitat taxonomic weights are shown in Figure 3. Cross-habitat results for error rate and Bray-Curtis dissimilarity were similar but are not shown. Note that larger F-measure is better and smaller error rate or Bray-Curtis dissimilarity is better. The total number of samples for each EMPO 3 habitat are shown in Table OM1. The results for bespoke and uniform weights are also summarised in Supplementary Tables 1-3.

Without exception, for every EMPO 3 habitat and all measures of classification accuracy at genus and species levels, bespoke weights always outperformed average weights, and average weights always outperformed uniform weights (Supplementary Table 1, Figure 2, Supplementary Figures 2-3. Across the 14 EMPO 3 habitats, average Bray-Curtis dissimilarities at species level were 0.126 (0.009 standard error), 0.15 (0.01 standard error), 0.23 (0.02 standard error), for bespoke, average, and uniform weights respectively. That is an almost two-fold increase from average bespoke weights Bray-Curtis dissimilarity to that for uniform weights. The average error rates were 14.3% (0.9% standard error), 16% (1% standard error), and 25% (2% standard error), again for bespoke, average, and uniform weights at species level.

The corresponding average F-measures were 0.887 (0.007 standard error), 0.871 (0.008 standard error), and 0.81 (0.02 standard error). Note that the variance of the uniform weights results across the EMPO 3 habitats was always greater than for bespoke or average weights. Across the EMPO 3 types, paired t-test differences between bespoke and average weights and average and uniform weights were significant in all cases; maximum P was 4.2×10^{-4}. Cross-habitat weights outcomes occupied a spread but rarely outperformed average weights (8 of 182 comparisons); however, cross-habitat weights frequently outperformed uniform weights (117 out of 182 comparisons) (Figure 3). Thus, it appears that uniform weights gravely misrepresent natural species distributions, marring classification accuracy. By comparison, any type of naturally derived taxonomic weight usually improved classification accuracy, even if those weights were derived from a dissimilar habitat type.

Using the cross-habitat weights it was possible to quantify the relationship between classification accuracy and taxonomic weight misspecification over 182 comparisons. We first calculated the differences between error rates using cross-habitat weights and the error rates using bespoke weights. We then calculated the corresponding Kullback-Leibler divergence between the bespoke and cross-habitat weights for each difference and discovered a significant correlation (Pearson $r^2 = 0.57$, $P < 2.2 \times 10^{-16}$, see Figure 4). We performed the same test with F-measure and discovered a negative correlation of roughly the same magnitude (Pearson $r^2 = 0.58$, $P < 2.2 \times 10^{-16}$). In our tests, using the bespoke weights yielded the best classification accuracy at every level, regardless of how we measured it. This result refines that finding to show that the amount by which performance degrades for taxonomic weights other than the default weights is proportional to how different they are to the bespoke weights. The implication is that any improvement of uniform weights in the direction of bespoke weights is worthwhile,

and that if bespoke weights are not available, then taxonomic weights from a similar habitat or the average weights should be used for classification.

We investigated what factors lead the classification accuracy for bespoke weights to vary between EMPO 3 habitats. To give an idea of the shapes of the taxonomic weights distributions, the cumulative distributions of taxonomic weights for each EMPO 3 habitat type are shown in Supplementary Figure 4. The distributions are qualitatively similar, with the most abundant 500 out of 5,403 species for each habitat accounting for greater than 93% of the mass in each case. While it is not shown in Supplementary Figure 4, it is also worth noting that the most common taxa for each habitat type are similar: the union of the sets of taxa that account for the first 95% of weights for each habitat type contains only 1,571 taxa.

We first examined whether the diversity of an EMPO 3 habitat was related to classification accuracy under bespoke weights. Regression of error rate against the entropy of the taxonomic weights for each of the 14 EMPO 3 habitats showed no significant relationship (Pearson r^2 = 0.12, P = 0.23).

The classification accuracy for a given EMPO 3 habitat type was instead found to be largely explained by specific interaction between taxonomic weights and the similarity among reference sequences. We discovered a significant correlation between the confusion index (see Methods) and the error rate using bespoke weights for each of the 14 EMPO 3 types (Pearson r^2 = 0.72, P = 1.3×10^{-4}) (Figure 5). A negative correlation of a similar magnitude was found for F-measure (Pearson r^2 = 0.63, P = 7.1×10^{-4}). While much has been written about the difficulty of establishing species-level identity from short read marker-gene sequences[1-5], to our knowledge

this is the first instance of a systematic quantitative analysis of how difficult this problem is in the light of which species co-occur. By using typical weights (as estimated from the data) we have shown that while it is possible to find many examples where a taxonomic classifier can be confused at species level if presented with isolated genetic sequences and the entire reference database, that in practice the problem does not have to be that hard. More than explaining variation between habitat types, these discoveries give a clear indication of the basis for the improvements that we see when using bespoke weights.

The error rate was tested for each of the phyla present in the observed taxa across all 14 EMPO 3 habitats under the assumptions of uniform and bespoke weights. The results for the most abundant phyla (those with average abundance > 0.5%) are shown in Supplementary Figure 5, where error rate and abundance was averaged over the habitat types. We observed that reads from some phyla are significantly more difficult to classify than others. For instance, using uniform weights, the error rate was 44% (0.7% standard error) for Firmicutes but 4% (0.2% standard error) for Acidobacteria. For the two most abundant phyla, Proteobacteria and Firmicutes, the decrease in incorrect classifications from uniform to bespoke weights was substantial, from 35% (0.7% standard error) to 22% (0.4% standard error) and from 44% (0.7% standard error) to 24% (0.3% standard error), respectively (maximum t-test $P = 8.4 \times 10^{-6}$). For Firmicutes that is an almost two-fold reduction in the number of incorrectly classified reads. These increases in accuracy underline the consistent increases in accuracy from uniform to bespoke weights that we have observed throughout this study.

Shotgun sequencing data, which has the potential to be less biased and higher-resolution than short amplicon sequences[6], may provide high-accuracy taxonomic weights to further increase

the value of high-throughput amplicon sequence data. To test this hypothesis, we downloaded 71 stool samples from the Human Microbiome Project website[7] for which shotgun and marker-gene data were available. Again using cross validation and treating the shotgun sequencing taxonomic classifications as ground-truth, the taxon detection rate (TDR)[1] for species-level classification of denoised 16S rRNA gene sequences improved from 0.46 (0.009 standard error) to 0.54 (0.01 standard error) when using shotgun-derived taxonomic weights relative to uniform weights (paired t-test $P = 1.4\times10^{-20}$). TDR using shotgun weights at species level also exceeded that using uniform weights at genus level (0.50, 0.009 standard error) (paired t-test $P = 3.9\times10^{-5}$).

Note that this is also a verification of our findings that does not use cross validation on reference sequences or the Greengenes[8] reference taxonomy.

It is clear that bespoke weights increase accuracy where uncertainty exists by favouring taxa that are more abundant for a given habitat. To test how this affects classification accuracy of rare species, we measured the qualitative measures Taxon Accuracy Rate (TAR)[1] and Taxon Detection Rate (TDR)[1], broken down by habitat. The results are shown in Supplementary Figures 7 and 8. TAR is the fraction of the observed taxa that were expected to be observed for a given sample. TDR is the fraction of taxa that were expected to be observed that were observed for a given sample. As they rely purely on presence and absence of taxa in a sample, they are less affected by species abundance. Across the 14 EMPO 3 habitats considered, the average TDR and TAR was better for bespoke weights than for uniform weights. This difference was significant at 5% significance for TDR (paired t-test $P = 2.2\times10^{-13}$) but not for TAR (paired

t-test $P = 0.5$). TDR followed the same trend shown in every other test that we performed and TAR was no worse for bespoke weights than it was for uniform weights.

We went further to search for whether rare species were being misclassified under the use of bespoke weights. In Supplementary Figure 9, several histograms represent the relationship between average species abundance and the difference in error rate between uniform and bespoke weights. A positive difference indicates that uniform weights outperformed bespoke weights for a given species on average across the 14 EMPO 3 habitats. Graphically, there is some evidence that the error rate degrades for bespoke weighted classifiers for species with average abundance of less than 10^{-4}. As the distribution is strongly peaked for no change in error rate for abundances of less than around 10^{-3}, we did not perform a linear regression. For a sample of 10,000 reads, that represents roughly one read attributable to that species. We concede that if rare species that one would expect to exist as singletons at usual sequencing depths are important to an experimental technique, then classification should be performed with uniform and bespoke classifiers.

Supplementary References

1. Bokulich, N. A. *et al.* Optimizing taxonomic classification of marker-gene amplicon sequences with QIIME 2's q2-feature-classifier plugin. *Microbiome* **6,** 90 (2018).
2. Cole, J., Konstantinidis, K, Farris, R. & Tiedje, J. Microbial diversity and phylogeny: extending from rRNAs to genomes. *Liu WT, Jansson JK (ed.)* **515,** 1–19 (2010).
3. Janda, J. M. & Abbott, S. L. 16S rRNA gene sequencing for bacterial identification in the diagnostic laboratory: pluses, perils, and pitfalls. *J. Clin. Microbiol.* **45,** 2761–2764 (2007).

4. Jovel, J. *et al.* Characterization of the Gut Microbiome Using 16S or Shotgun Metagenomics. *Front. Microbiol.* **7** (2016).
5. Edgar, R. C. Accuracy of taxonomy prediction for 16S rRNA and fungal ITS sequences. *PeerJ* **6,** e4652 (2018).
6. Segata, N. *et al.* Metagenomic microbial community profiling using unique clade-specific marker genes. *Nat. Methods* **9,** 811 (2012).
7. Huttenhower, C. *et al.* Structure, function and diversity of the healthy human microbiome. *Nature* **486,** 207 (2012).
8. McDonald, D. *et al.* An improved Greengenes taxonomy with explicit ranks for ecological and evolutionary analyses of bacteria and archaea. *ISME J.* **6,** 610 (2012).
