# Supplementary material for: Species abundance information improves sequence taxonomy classification accuracy
Source: Nat Commun. 2019 Oct 11;10:4643. doi: 10.1038/s41467-019-12669-6 (PMC6789115; doi:10.1038/s41467-019-12669-6)
Supplement: Supplementary file 3 — Reporting Summary [file 41467_2019_12669_MOESM3_ESM.pdf]

nature research

Corresponding author(s): Gavin A Huttley
Last updated by author(s): Sep 11, 2019

Reporting Summary

Nature Research wishes to improve the reproducibility of the work that we publish. This form provides structure for consistency and transparency in reporting. For further information on Nature Research policies, see Authors & Referees and the Editorial Policy Checklist .

Statistics

For all statistical analyses, confirm that the following items are present in the figure legend, table legend, main text, or Methods section.

n/a	Confirmed	
☐	☒	The exact sample size (*n*) for each experimental group/condition, given as a discrete number and unit of measurement
☐	☒	A statement on whether measurements were taken from distinct samples or whether the same sample was measured repeatedly
☐	☒	The statistical test(s) used AND whether they are one- or two-sided<br>*Only common tests should be described solely by name; describe more complex techniques in the Methods section.*
☐	☒	A description of all covariates tested
☐	☒	A description of any assumptions or corrections, such as tests of normality and adjustment for multiple comparisons
☐	☒	A full description of the statistical parameters including central tendency (e.g. means) or other basic estimates (e.g. regression coefficient) AND variation (e.g. standard deviation) or associated estimates of uncertainty (e.g. confidence intervals)
☐	☒	For null hypothesis testing, the test statistic (e.g. *F*, *t*, *r*) with confidence intervals, effect sizes, degrees of freedom and *P* value noted<br>*Give P values as exact values whenever suitable.*
☒	☐	For Bayesian analysis, information on the choice of priors and Markov chain Monte Carlo settings
☐	☒	For hierarchical and complex designs, identification of the appropriate level for tests and full reporting of outcomes
☐	☒	Estimates of effect sizes (e.g. Cohen's *d*, Pearson's *r*), indicating how they were calculated

Our web collection on statistics for biologists contains articles on many of the points above.

Software and code

Policy information about availability of computer code

Data collection	Data was collected using q2-clawback version 0.0.2 (https://github.com/BenKaehler/q2-clawback) and redbiom version 0.1.0 (https://github.com/biocore/redbiom). Data was also downloaded from NCBI and the Human Microbiome Project websites. Please see references and details in the manuscript.
Data analysis	Analysis was performed using q2-clawback version 0.0.5 (https://github.com/BenKaehler/q2-clawback) (also available through conda and pip) and paycheck version 0.0.3 (https://github.com/BenKaehler/paycheck).

For manuscripts utilizing custom algorithms or software that are central to the research but not yet described in published literature, software must be made available to editors/reviewers. We strongly encourage code deposition in a community repository (e.g. GitHub). See the Nature Research guidelines for submitting code & software for further information.

Data

Policy information about availability of data

All manuscripts must include a data availability statement. This statement should provide the following information, where applicable:
- Accession codes, unique identifiers, or web links for publicly available datasets
- A list of figures that have associated raw data
- A description of any restrictions on data availability

All of the raw data is available from https://doi.org/10.5281/zenodo.2548899 and https://doi.org/10.5281/zenodo.2549777.

Field-specific reporting

Please select the one below that is the best fit for your research. If you are not sure, read the appropriate sections before making your selection.

Life sciences study design

All studies must disclose on these points even when the disclosure is negative.

Sample size	All available data was downloaded from Qiita (https://qiita.ucsd.edu/) for the 14 EMPO 3 habitat types included in the study. For the HMP samples that were analysed, all samples for which shotgun and amplicon data were available were analysed.
Data exclusions	The three EMPO 1 control EMPO 3 habitat types were excluded, as well as Hypersaline (saline), Aerosol (non-saline), and Plant surface, which all had fewer than nine samples in the Qiita database. The smallest EMPO 3 data set that was included in the study had 152 samples. As 5-fold cross-validation was performed on the samples, it was determined that nine and fewer samples were too few for meaningful results. In no instance did results influence our decision to exclude data.
Replication	Findings were similar across the 14 different EMPO 3 habitat types. Differences between the results for each EMPO 3 habitat type are analysed in some detail in the manuscript, including relationships with other properties of the data.
Randomization	For each EMPO 3 habitat type, samples were randomly allocated into five folds for cross validation. The 16S reference sequences were stratified by taxonomy and randomly allocated into five folds for cross validation. Details are given in the Online Methods.
Blinding	This work is a comparison of methods for taxonomic classification. Each method was tested using the same code on the same data sets. There was no opportunity for investigators to alter the outcomes between experiments.

Reporting for specific materials, systems and methods

We require information from authors about some types of materials, experimental systems and methods used in many studies. Here, indicate whether each material, system or method listed is relevant to your study. If you are not sure if a list item applies to your research, read the appropriate section before selecting a response.

Materials & experimental systems

n/a	Involved in the study	
☒	☐	Antibodies
☒	☐	Eukaryotic cell lines
☒	☐	Palaeontology
☐	☒	Animals and other organisms
☒	☐	Human research participants
☒	☐	Clinical data

Methods

n/a	Involved in the study	
☒	☐	ChIP-seq
☒	☐	Flow cytometry
☒	☐	MRI-based neuroimaging

Animals and other organisms

Policy information about studies involving animals; ARRIVE guidelines recommended for reporting animal research

Laboratory animals	The study did not involve laboratory animals.
Wild animals	The study did not involve and wild animals.
Field-collected samples	The study only used microbial data that is freely available in online databases. Where those samples were field-collected, details are available in those studies or in those databases. Details are provided in the manuscript.
Ethics oversight	This study only used microbial data that is freely available in online databases. No ethics oversight was required.

Note that full information on the approval of the study protocol must also be provided in the manuscript.
